# Environmental drivers of size-based population structure, sexual maturity and fecundity: A study of the invasive blue crab *Callinectes sapidus* (Rathbun, 1896) in the Mediterranean Sea

**DOI:** 10.1371/journal.pone.0289611

**Published:** 2023-08-07

**Authors:** Guillaume Marchessaux, Vojsava Gjoni, Gianluca Sarà

**Affiliations:** 1 Laboratory of Ecology, Department of Earth and Marine Science (DiSTeM), University of Palermo, Palermo, Italy; 2 NBFC, National Biodiversity Future Center, Palermo, Palermo, Italy; 3 Department of Biology, University of South Dakota, Vermillion, SD, United States of America; University of Split, Faculty of science, CROATIA

## Abstract

The blue crab *Callinectes sapidus* (Rathbun, 1896) is one of the most invasive species in the Mediterranean Sea. Understand how the populations are maintained and how the environment is driving the populations in the areas invaded is the key to an effective future management. This current study is presenting a monthly long-term monitoring of the blue crabs’ population structure, body size, sexual maturity, reproduction periods and fecundity, and their relationships with environmental factors in a saltmarshes system in Italy. During winter, high densities (15 ± 8 ind m^-2^) of early juveniles (< 2 cm) were observed, and their numbers decreased due the population growth until summer. The size-spectra showed that across different temperature (18–26°C) and salinity (24–40 psu) gradients, the growth period for males is faster than for females. Based on abdominal analysis, sexual maturity was defined at ∼12 cm for males and females but the population was in favor of males that were more than 66% of the time mature throughout the year. Copulations periods were identified between spring and autumn when more than 50% of females were matures, and ovigerous females’ migrations were observed in late summer. Our study expand our understanding of how the environment interacts to effect physiological and biological processes of alien species and improve our ability to make predictions of how environmental change the distribution of the alien species in the future. Based on our results, we also discuss which population control strategy would be most effective based on the data available in the literature.

## Introduction

An important point in ecology is to understand how environmental factors are affecting species distribution and population structure [1] which could be profoundly affected by disturbance (*sensu* Sousa [2]). This understanding involves study the population size and dynamics, reproduction strategy and species ability to adapt to environmental changes [3]. One of the main factor to consider is the species body size which is one of the main performance traits having a strong potential to determine the negative impact of environmental factors on the composition of local populations [4]. Studying the population size-spectra and how the environmental factors are able to shape the size based population structures, constitutes a powerful indicator of marine ecosystems status [5]. In the case of non-indigenous species (NIS) this approach proposes new monitoring descriptors such as (i) the quantification of ability of NIS to adapt in new habitats and (ii) the understanding of how environmental factors are able to address the population size structure, dynamics and biology.

The blue crab *Callinectes sapidus* Rathbun (1896), a portunid native from the Atlantic coasts of America (from Nova Scotia in the north to Argentina), was recorded in the Mediterranean Sea in 1949 and the Black Sea and the Sea of Azov in 1967 [6]. The species is particularly fertile [7, 8] and tolerates a large range of temperatures [9] which contribute to its invasion, expansion and maintenance success in the Mediterranean in the last decades [10]. *Callinectes sapidus* is widely distributed in the Mediterranean Sea along the Northern (from Spain to Turkey) and southern (Egypt, Tunisia, Algeria and Morocco) coasts [11]. This species is an opportunist predator [12, 13] with a particular aggressive behavior [14] inducing strong impacts on native biodiversity (competition with autochthonous species, local species extinctions) and artisanal fisheries (net destruction, fish mutilations) [15]. It’s now accepted that the only way to control the species is to fish it and use it as a resource. In its native range, blue crab fisheries are recognized for their commercial value and catch volume, making the blue crab fishery one of the most important fisheries on the USA east coast [16]. Blue crab is of major fishing interest, consumed in large quantities, mainly in the USA and Mexico [17, 18]. In some Mediterranean regions (e.g. Adriatic Sea; Egypt; Turkey; Tunisia, Spain), the blue crab fishery is used as a population control measure, and its use and sale has made it possible to compensate for the economic losses suffered by the artisanal fishery [19, 20].

Due to its complex life cycle—comprising numerous larval (zoeae), post-larval (megalopae), and juvenile phases punctuated by an abrupt transition to adults [14]–*C*. *sapidus* occurs in various habitats and salinity gradients (brackish and sea waters) [21]. Reproduction between males and females takes place in low salinity waters, and female crabs migrate to polyhaline zones to produce and incubate eggs [22]. The adult males remain in the low salinity waters [23]. Once the eggs mature, the females migrate to the sea (salinity up to 30) to release the planktonic zoeae larvae [24]. After a long transport in the sea, the zoeae larvae differentiate into megalops that gradually return to the desalinated areas (e.g. lagoons, estuaries, saltmarsh, etc.) which play a role of nursery [24, 25]. Juvenile crabs use a diverse range of nurseries throughout ontogeny, mainly in structurally complex habitats (dense aquatic vegetation) for individuals smaller than 25 mm carapace width [26–30], then non-vegetated secondary nurseries with a sandy-muddy substrate and low salinity for individuals > 25 mm [25, 31]. The juvenile blue crabs stay in these sandy-muddy substrate until they reach sexual maturity at about 100–200 mm body weight (age at maturity and longevity ~ 1–2 years and 3 years, respectively, depending on phenology and geographic location) [23, 32]. The complex blue crabs life cycle present currently a barrier to the possible control measures and some answers are urgent about when and how to implement an effective control measure. Thus, studying the blue crabs life cycle, especially the population growth could be the key to define the sensitives periods where control measures could be implemented.

In the Mediterranean Sea, *C*. *sapidus* is found in various habitats (river mouths, brackish lagoons, on the coast) but patterns of blue crab population size structure and maturity stages in these diverse regions are still poorly understand. The land structure geography of Sicily does not offer favorable desalinated habitats (e.g. estuaries, lagoons) preferred for the blue crabs’ life-cycle. However, the high frequency of sightings and occurrences of *C*. *sapidus* in Sicily [33] suggests that they find favorable conditions for maintaining sustainable populations. Following the numerous reports of *C*. *sapidus* by fishermen on the western Sicilian coasts, an investigation was conducted to identify viable populations. In July 2021, a population of blue crabs was found in the heart of the natural reserve of Trapani ("Saline di Trapani e Paceco") which includes a large complex of human-made ponds hosting important aggregations of saltmarshes [34, 35] with some currently exploited for salt and others in restauration.

Shallow subtidal areas of saltmarsh are known to be inhabited by the blue crab *C*. *sapidus* in their native areas [36–39]. Although blue crabs have been reported to move into the intertidal zone of polyhaline [40, 41] and oligohaline waters [42], there are few data on the extent of intertidal salt marshes used by this large decapod crustacean in the Mediterranean Sea. These microhabitats are known to be refuge areas for *C*. *sapidus* where it found protection and high food quantities to sustain growth and reproduction [27, 31, 43, 44]. Even if *C*. *sapidus* is widespread in the Mediterranean Sea, little information exists on population structure, body size, sexual maturity, reproduction periods and growth. Few studies show the blue crab populations dynamics in the Mediterranean. Even if some efforts are performed to quantify the population size structure, data are missing on the males and females size at maturity, population growth and juveniles (< 2 cm) abundance, and, more importantly, the effect of environmental drivers conditioning the blue crab populations.

In the case of the blue crab, which is a generalist species with an important capacity of adaptation and larval and adult stages dispersal, using such descriptors allows to quantify the environmental key-factors inducing blue crab’s population size structure well-being in order to implement adaptative and effective management plans (e.g. population control). Salinity and temperature gradients are the major environmental factors that affect the metabolism [9] and the life cycle of *C*. *sapidus* [18]. In Sicily, the pronounced temperature and salinity oscillations in the Trapani saltmarshes (Italy) may have favored the establishment of *C*. *sapidus*. However, to understand population dynamics, in addition to allometric relationships, it becomes necessary to follow seasonal size spectra, sexual maturity and reproductive events, establishing migration routes and recruitment periods in saltmarshes.

Considering the above information, here we show a study case on how monitoring and inspecting blue crabs *C*. *sapidus* performance traits (population structure, body size, sexual maturity, copulation periods and fecundity) in relationships with environmental factors can assist practitioners in addressing effective NIS management measures in in the Mediterranean Sea.

## Materials and methods

### Study site and sampling strategy

The study site (named “Salina Grande”; 37°57’13.7"N, 12°29’51.3"E) is located in the Nature Reserve "Saline di Trapani e Paceco” (WWF Italy; SIC: ITA01007), Sicily (Italy) [34]. The reserve (area of 960 ha) consists of a plain characterized by a sandy coast and a large wetland (80% of the area). The remainder of the area is divided between areas of intensive human activity (10%), wooded and shrubby areas (5%), and agricultural areas (5%). Wetlands are represented by the following categories: reed thickets, ponds (30 ha) and salt marshes (750 ha). It is an important wetland used as a resting place by thousands of migratory birds and characterized by a purely halophilic environment of great naturalistic value. The vast wetland has different habitats such as natural mudflats, salt marshes, canals that are home to over 200 species of birds.

**Fig 1:**
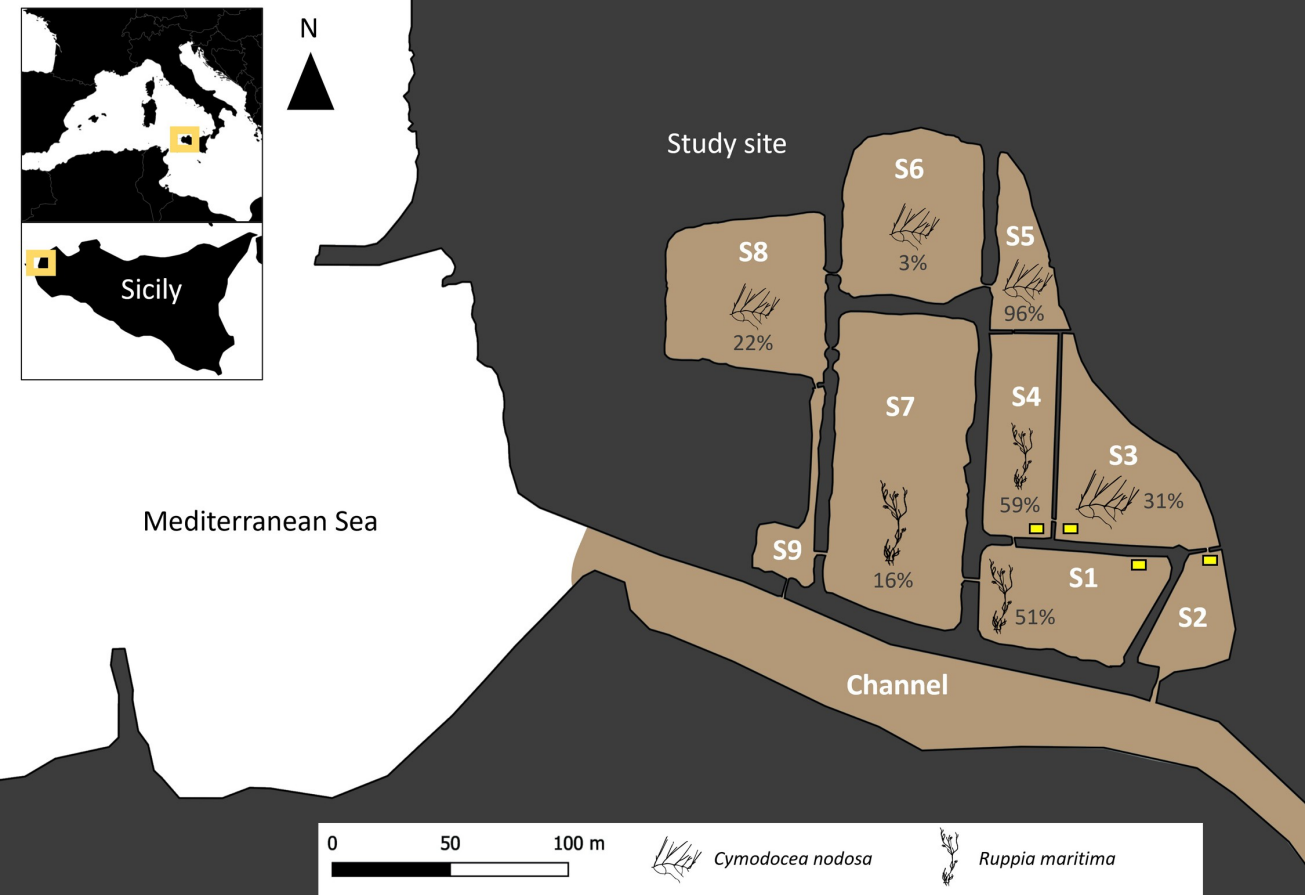
Map of the study site in the natural reserve of Trapani (Sicily). The yellow squares represent the position of blue crab traps. The white numbers correspond to the ID of each saltmarsh, the percentages represent the percentage of coverage by seagrasses, and the brown color represents the sandy-muddy substrate. Copyright: @Marchessaux, CC BY 4.0 license.

The study site includes a complex of 9 saltmarshes (max depth: ~ 40 cm) with 2 connections to the sea though a channel (Fig 1). We identified 3 substrate types from the field observations: sandy-muddy, *Cymodocea nodosa*, and *Ruppia maritima* [45, 46] (Fig 1 and S1 Table): three with only sandy-muddy substrate, six with sandy-muddy and *Ruppia;* and only one with sandy-muddy and *Cymodocea* substrates. The channel connecting the saltmarsh system and the sea was only composed by a sandy-muddy substrate. The first blue crab, *C*. *sapidus*, was observed on 20^th^ July 2021. Since, a monitoring was performed for 18 months. A total of 49 sampling campaigns were carried out between July 2021 and December 2022. This study was performed in collaboration with the WWF Italy, manager of the "Saline di Trapani e Paceco” natural reserve, they give to us the permission to perform sampling and environmental study in this site.

Four traps (100 cm x 80 cm; 4 apertures of 30 cm) specifically designed for this study (S1 Fig) were used to sample blue crabs. The duration of the traps immersion was 5 days using chicken pieces as a bait. A minimum of 30 blue crab organisms were sampled in the 4 saltmarshes, and placed in a cooler. Due to the small size of the study site, the total specimen number across all 4 saltmarshes was considered for the study. At the beginning of our study, the northern salt marshes (S5 to S9, Fig 1) were disconnected from the others by wooden gates and no blue crabs were observed in. However, after a storm during autumn 2022, they are currently connected to the rest of the system. In order to maintain consistency in our study, we have therefore chosen not to include these northern salt marshes.

During the survey blue crab early stages (< 2 cm) and juveniles (2–5 cm) were observed in samples of sand-mud performed in the saltmarsh S3. To sample them and to quantify their local density, 10 replicates of 40 x 40 cm quadrats were performed in S3 at 1 m from the edge of the basin. The content of the quadrat was collected with a hand net and was gently rinsed with saltmarsh water on a 500 μm mesh. Juveniles of blue crabs were gently picked up by hand and conserved in a 1 L plastic bottle with saltmarsh water for biometry measurements. The density of juveniles was reported per square meter and monthly averaged (± standard deviation).

To determine the natural behavior of the organisms, the monitoring operators walked along the edges of all the saltmarshes making 10 round trips to observe the organisms. The shallow depth (< 40 cm) allowed for easy observation of the individuals. During this visual monitoring, the operators reported the presence of couples of blue crabs in reproduction (male and female abdomen against abdomen). This information made it possible to determine the reproduction periods in the study site.

Temperature was recorded continuously in each pond every hour using a HOBO Pendant® loggers (mod. MX2201, ± 0.5°C accuracy), while salinity was measured for each sampling date using a multiparameter probe (HANNA® HI 98194, ± 0.1 unity accuracy).

### Size structure, sexual maturity and females fecundity

**Fig 2:**
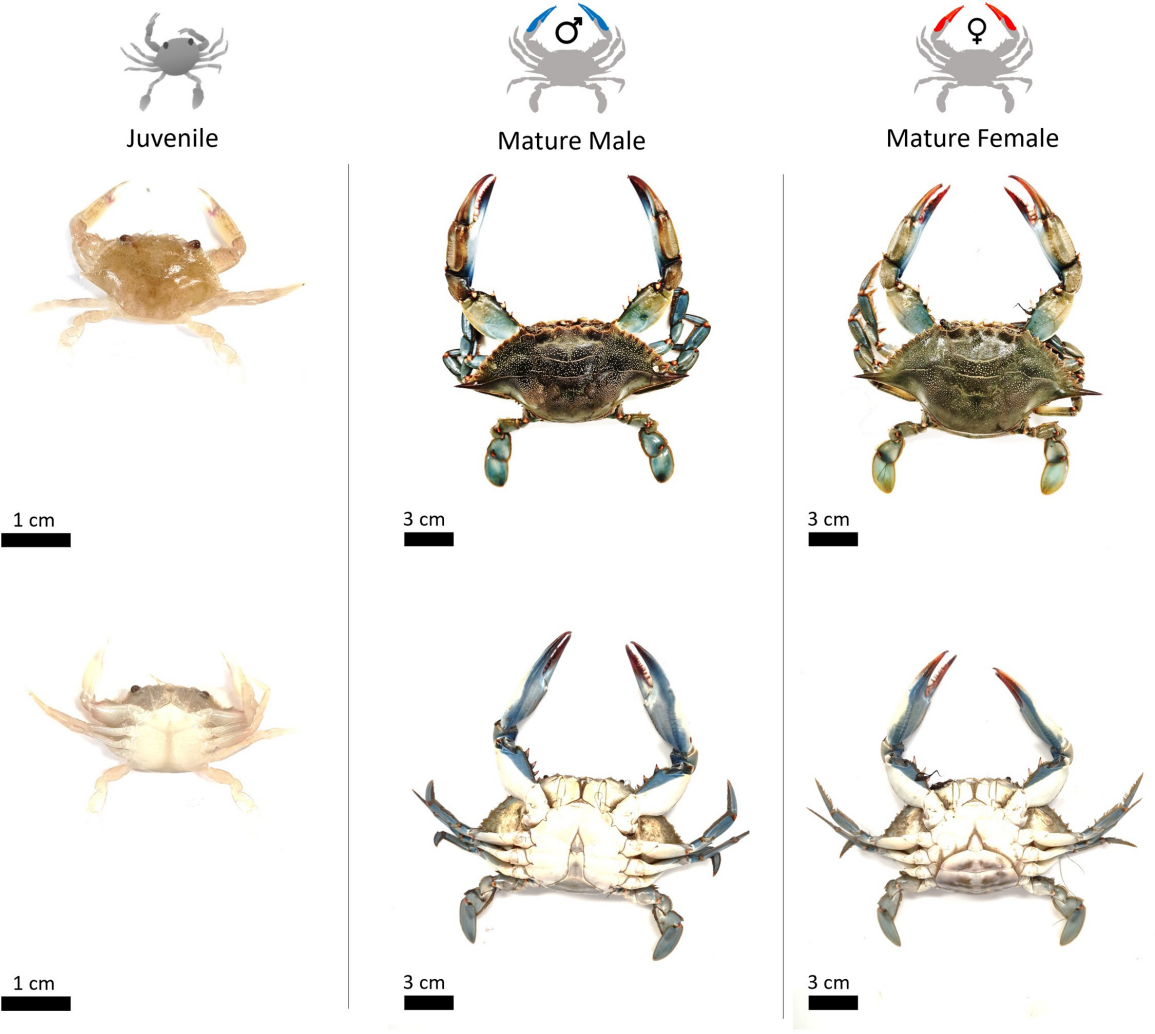
Pictures of the juveniles, matures males and females *Callinectes sapidus* sampled in the saltmarshes of Trapani (Italy); dorsal and ventral side. Photos: G. Marchessaux. Copyright: @Marchessaux, CC BY 4.0 license.

In laboratory each blue crab individual was sexed and the maturity determined (immature or mature) based on the abdomen morphological analysis (Fig 2). The maturity of blue crab female was determined by the shape of the abdomen: a triangular abdomen was considered immature if it was sealed and the vulvas were indistinct. The mature adult female has the characteristic enlarged, heavily pigmented and fully expandable abdomen and pleopods [14, 47]. For males, organisms were immature if the abdomen was closed and/or partially locked to the sternum with the penis inserted into the gonopods [18, 48–50]. A mature adult male showed a free and fully expandable abdomen (Fig 2).

For all organisms with size up to 5 cm, the carapace width (CW, distance between the two dorsal spines) and the abdomen width (ABD) were measured using a graduate ruler (± 1 mm accuracy). The wet weight (WW) of each individual was measured using a digital balance (± 0.1 g accuracy). For the smaller organisms (< 5 cm), CW and ABD were measured using the software ImageJ (± 0.1 mm accuracy) after taking a picture of the dorsal and ventral sides with a stereomicroscope Zeiss Axio Zoom V16. WW was measured using a digital microbalance Metro Toledo EL104 (± 0.0001 g accuracy).

For ovigerous females, the egg masses were carefully removed from pleopods and their wet weight (± 0.1 g accuracy) was measured using the digital microbalance. The embryotic development was determined on the basis of the eggs’ color [51–53]: I-initial (yellow), II-intermediate (orange), and III-pre-hatching (dark brown). In a second time, eggs were separated in a 5% sodium hypochlorite solution [52]. The total eggs samples were separated in two homogeneous partitions and one was counted in a Dollfuss cuvette under the stereomicroscope. 1 000 eggs were measured using the software ImageJ (± 0.1 μm accuracy) after taking pictures with a stereomicroscope.

### Data analysis

The length-weight (CW-WW) allometric relationship was determined by an exponential equation for males and females respectively, and the males/females abdomen-CW relationship was determined by a linear regression. Size distribution (frequency for every 1 cm) for males and females was seasonally calculated and the Gaussian regressions determined. The seasons were determined as: winter: January-Mars; spring: April-June; summer: July-September; autumn: October-December. For each month, the allometric CW-WW regressions were performed for both sexes and the slopes, after linearization, were extracted. Using a 3-dimensional plot, slopes were represented as a function of temperature and salinity to determine the males and females growth.

To follow the population goodness condition, the Fulton’s condition factor (K) was monthly calculated following the equation (1): K = (W/L^3^)*100 where W and L are respectively the total weight and carapace width [54]. 3-dimensional plots were performed to correlate the Fulton’s condition factor with temperature and salinity for both sexes and as a function of the maturity stage (immature; mature).

The monthly percentage of immature and mature males and females was calculated in order to quantify the sexual population structure. The monthly sex ratio (Males/Females) was estimated. The length at first maturity of females and males (the length at which 50% of the organisms had become mature) was determined from the relationship between the percentages of mature crab (males and females separately) and the CW classes of 1 cm. The proportion (P) of sexually mature of CW (males and females) was fitted to the logistic equation (3): $P=\frac{1}{\left(1+exp^{\left[-r\left(L-L_{m}\right)\right]}\right)}$ which in straight line form is: $\ln\left[\frac{1-P}{P}\right]=rL_{m}-rL$ where r (-b) is the slope of the curve and L_m_ is the mean length at sexual maturity on the CW which corresponds to a proportion of 50%. Size at sexual maturity (L_m_) was calculated from -(a/b). A logistic function was fitted to the proportion of mature males/females. All plots and regressions were performed using the software SigmaPlot 12.5.

To test the differences between the eggs’ diameter at the different embryotic development stages identified, an ANOVA and a Bonferroni post-hoc test (significant difference = p < 0.05) were performed using R Studio (version 2021.09.0). ANOVA was also used to identify the differences between matures males and females periods.

## Results

**Fig 3:**
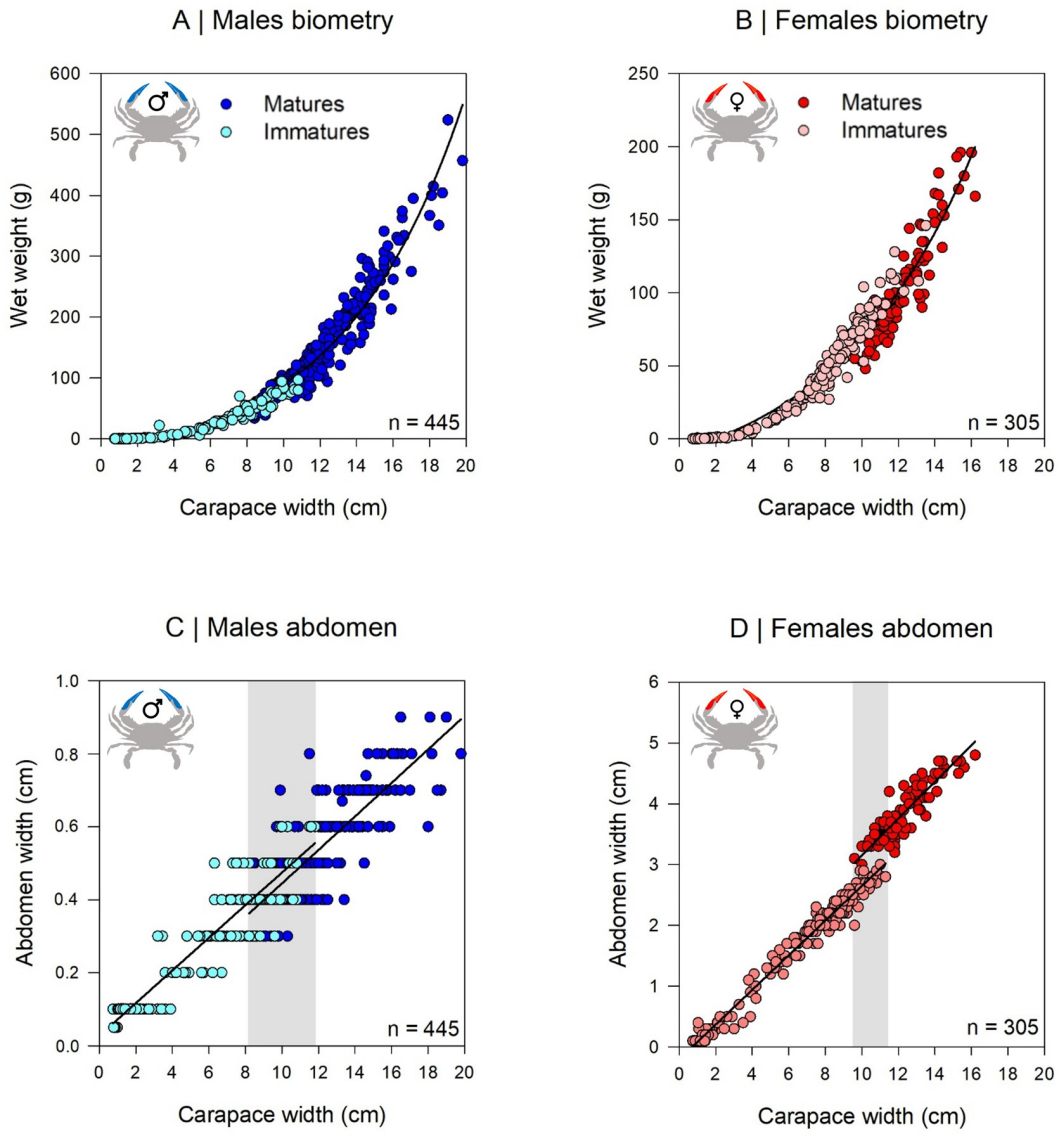
Relationship between carapace width (CW) and wet weight (WW) for (A) males and (B) females; relationship between CW and abdomen width for (C) males and (D) females. Copyright: @Marchessaux, CC BY 4.0 license.

A total of 750 blue crabs (445 males and 305 females) were evaluated for size-length characteristics and sexual maturity based on their morphology. The size distribution of immatures and matures males and females ranged for males from 0.75 to 10.8 cm (immatures) and from 8.2 to 19.8 cm (matures); for females from 0.74 to 10.4 (immatures) and from 9.6 to 16.2 (matures) (Fig 3A and 3B and Table 1A).

**Table 1:** (A) Width (CW) and weight (WW) characteristics (mean, minimum (Min.), maximum (Max.)) for the blue crab caught in the saltmarshes of Trapani (Italy). (SE = standard error); (B) allometric parameters of the relationship (WW = a*exp(b*CW) + y0) between the carapace width (CW) and the weight (WW) and (C) allometric parameters of the relationship (ABD = a*CW + b) between the abdomen width (ADB) and the carapace width (CW). *: significant difference.

**A**							CW characteristics (cm)									WW characteristics (g)									
Sex	Maturity			n			Mean		SE			Min.		Max.		Mean			SE			Min.			Max.
Males	Immatures Matures			168 277			5.02 12.06		± 3.26 ± 2.29			0.75 8.20		10.80 19.80		25.16 152.06			± 27.18 ± 83.50			0.02 34.00			97.00 524.00
Females	Immatures Matures			205 100			6.52 12.34		± 3.02 ± 1.48			0.74 9.60		10.40 16.20		36.67 105.30			± 25.61 ± 35.93			0.02 48.00			104.00 196.00
**B**											Parameters of CW-WW relationship WW = a*exp(b*CW) + y0														
Sex		Maturity			n			a			b				SE(b)			R^2^			p value			y0	
Males		Immatures Matures Both			168 277 445			11.32 106.42 29.56			0.21 0.09 0.15				0.013 0.010 0.004			0.96 0.93 0.95			< 0.0001* < 0.0001* < 0.0001*			-15.63 -188.33 -45.17	
Females		Immatures Matures Both			205 100 305			18.43 138.66 29.69			0.17 0.07 0.13				0.009 0.053 0.007			0.95 0.83 0.94			< 0.0001* 0.2082 < 0.0001*			-25.99 -214.60 -38.20	
**C**										Parameters of ABD-CW relationship															
Sex			Maturity			n				a			b				SE(a)			R^2^			p value		
Males			Immatures Matures Both			168 277 445				0.045 0.046 0.043			0.027 -0.014 0.028				0.001 0.002 0.001			0.84 0.66 0.88			< 0.0001* < 0.0001* < 0.0001*		
Females			Immatures Matures Both			205 100 305				0.285 0.301 0.333			-0.199 0.139 -0.424				0.003 0.017 0.004			0.98 0.76 0.96			< 0.0001* < 0.0001* < 0.0001*		

The length-weight relationships (CW-WW) followed an exponential curve for both sex (Fig 3A and 3B and Table 1B). The slope coefficient *b* values were similar for both sex maturity stage: immatures males: 0.21, immatures females: 0.17; matures males: 0.09, matures females: 0.07 (Table 1B). The male and female abdomen sizes showed different linear regressions depending of the sexual maturity (Fig 3C and 3D and Table 1C); with a marked separation between immatures and matures females in contrast to males where the slopes were almost identical. For males, immatures/matures abdomen size was not clearly separated and both immature and mature organisms were observed between 8 and 10 cm (Fig 3C). For females, the separation was more marked with only an overlap of the two stages of sexual maturity observed between 9.9 and 11.3 cm (Fig 3D).

Temperature and salinity showed a typical Mediterranean seasonal trend (S2A Fig). The monthly temperature ranged from 11.9 ± 1.7°C (Jan. 2022) to 30.6 ± 2.1°C in July; with lowest temperature value at 7.6°C recorded on 26th January 2022 and highest at 35.9°C on 20^th^ July 2022. Salinity oscillated seasonally from 15 (17^th^ Feb. 2021) to 42 (6^th^ Aug. 2022) between winter and summer (S2B Fig).

**Fig 4:**
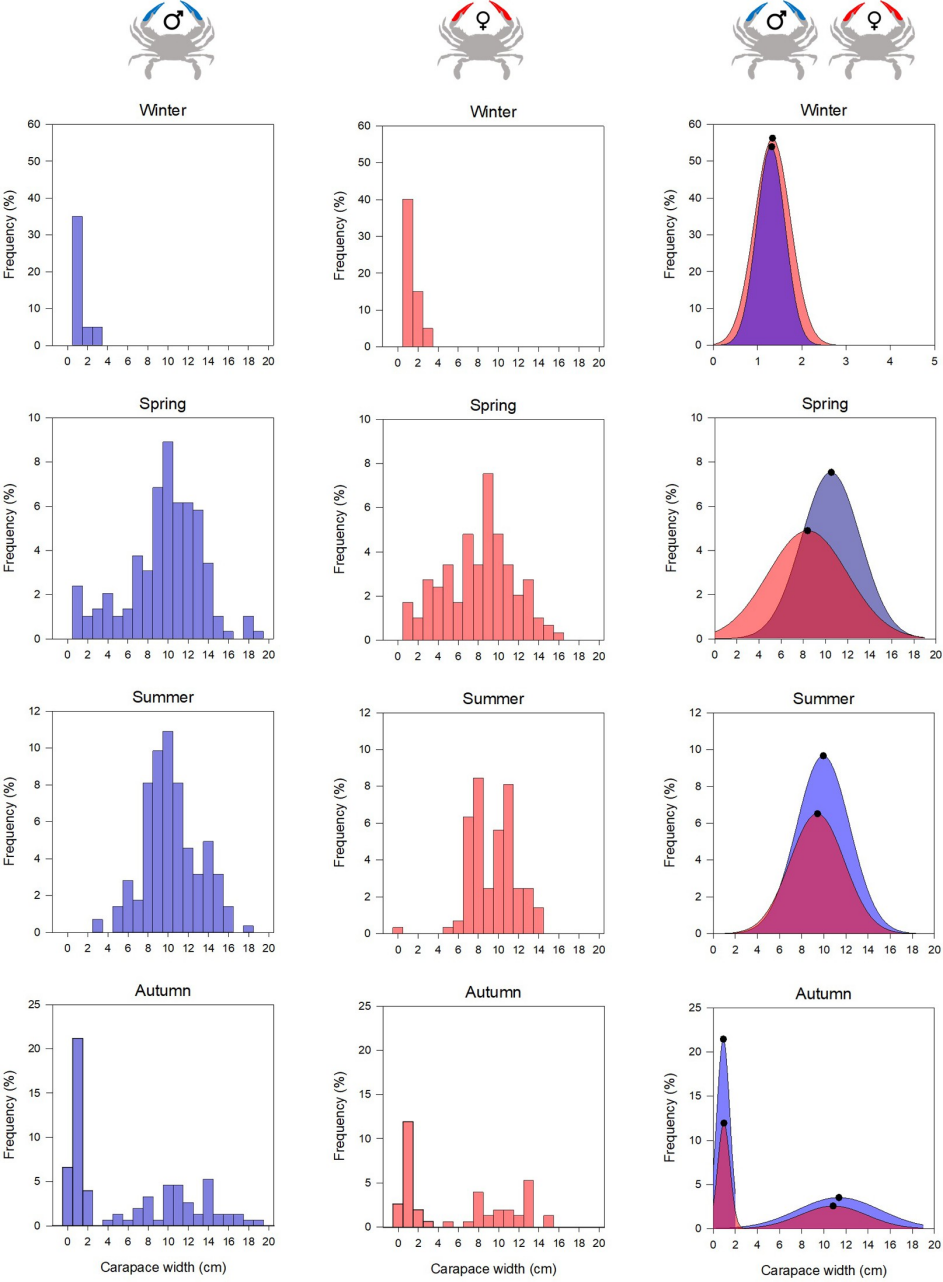
Seasonal blue crab size distribution of males (on the left, in blue), females (in the center, in red) and the estimated Kernel densities of each sex (on the right, the black dots represent the size with the highest frequency). Copyright: @Marchessaux, CC BY 4.0 license.

The size distribution of males and females showed a seasonal pattern (Fig 4). During winter only the early stages (< 3 cm) were founded with a major contribution (> 40% of 0–1 cm size class for both sexes. Both sexes size distribution was overlapped (Fig 4). Both sexes grew rapidly during spring to reach a dominant size at 10.6 cm for males and 8.4 cm for females. On the other hand, males showed larger size ranged (from 1 to 19.8 cm) than females (1 to 16 cm) inducing a lag of size distributions which were 86% overlapped (Fig 4). During summer, the both sexes showed the same sizes ranges with, for both, the same dominant sizes (9.9 cm for males; 9.4 cm for females) with both size distributions overlapped (Fig 4). Autumn was the season regrouping two distinct cohorts: the adult one with dominant contribution of 11.3 cm and 10.8 cm size for males and females respectively; and the juvenile cohort mainly composed by 0.9 cm organisms for both sexes (Fig 4).

The early stage (< 2 cm) and juveniles (< 5 cm) densities showed a seasonal pattern (S3 Fig). They were observed in March 2022 and their density decreased from 6 ± 3 ind m^-2^ to 2 ± 3.0 ind m^-2^ in May. Early stage and juveniles were absent in the samples until September where low density was measured (1 ± 3 ind m^-2^). Whereas a large arrival of early stage was recorded between November (15 ± 8 ind m^-2^) and December (12 ± 5 ind m^-2^).

**Fig 5:**
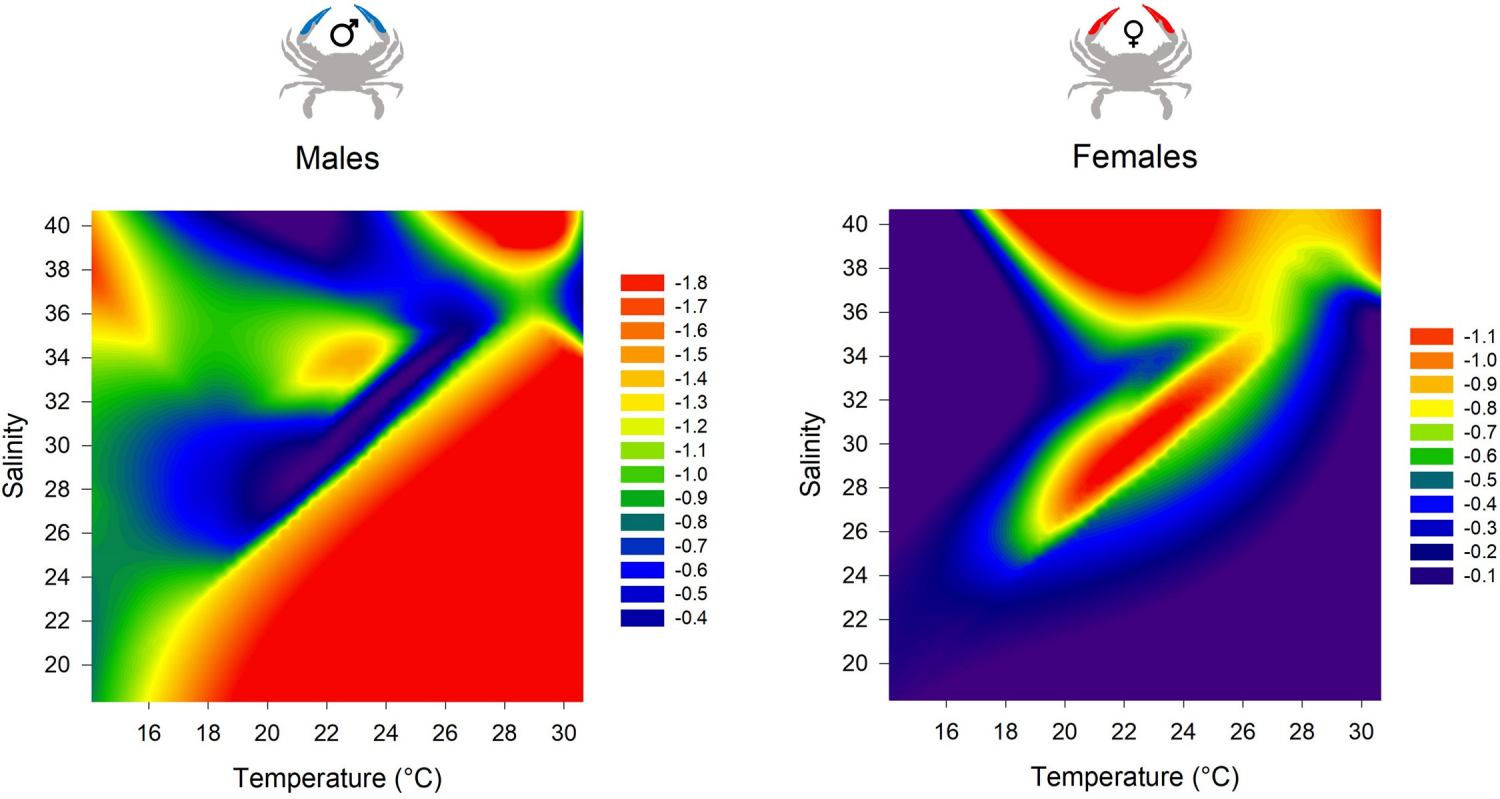
3-dimensional relationships of the monthly males and females CW-WW slope coefficients (a) as a function of temperature and salinity. Copyright: @Marchessaux, CC BY 4.0 license.

The relationship between temperature, salinity and the CW-WW slope coefficient as a proxy of growth change showed opposite results between males and females (Fig 5). For both sex, temperature had a positive effect on growth: higher the temperature, the larger the growth. Salinity had an opposite effect for males and females: the growth was higher for male for a large salinity range (16 units; from 24 to 40) than females where the highest growth values were observed on a smaller salinity range (12 units; from 28 to 40) (Fig 5). In other words, the size spectra showed that across different temperature and salinity gradients, the growth period for males is faster than for females (Fig 5).

**Fig 6:**
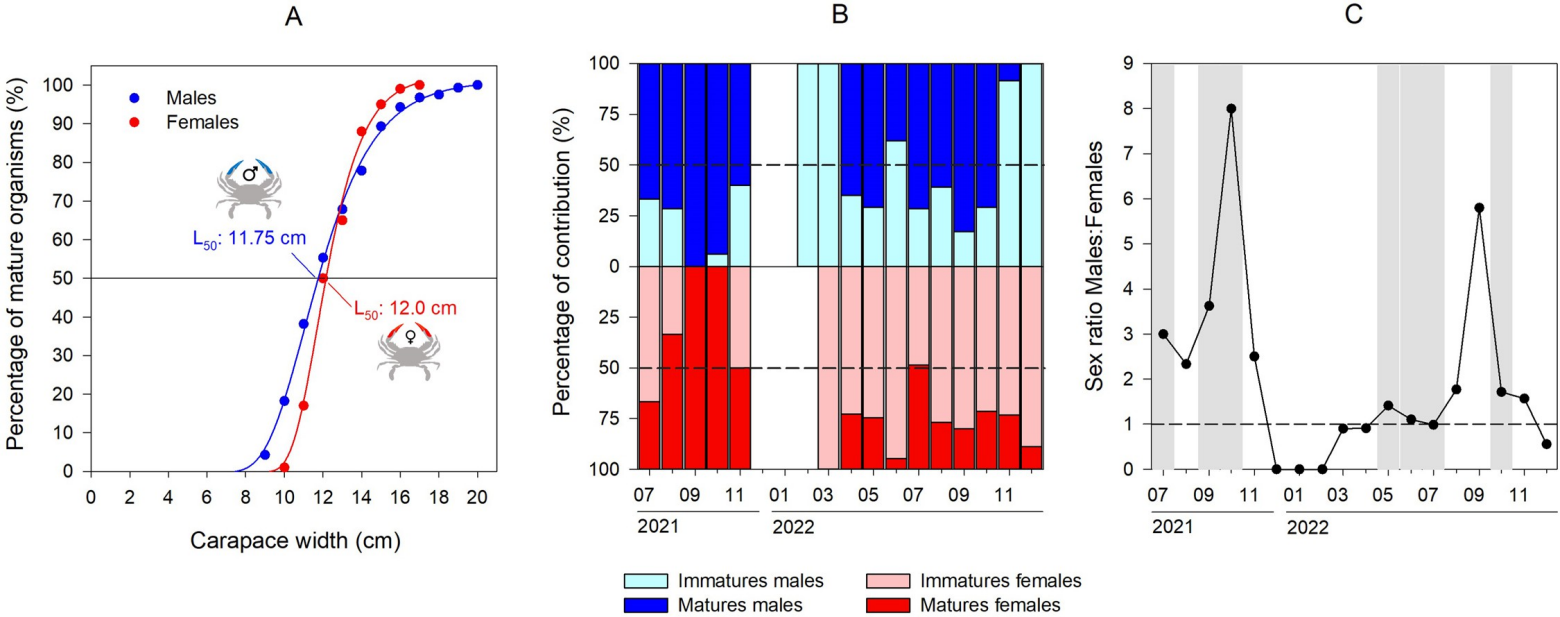
(A) Logistic regressions (solid lines) fitted to the percentage of mature males and females based on the carapace width (CW), L_50_ values represent the CW when 50% of the population was mature; (B) temporal evolution of the percentage of contribution of the immatures/matures males and females between July 2021 and December 2022; (C) temporal evolution of the sex ratio Males/Females between July 2021 and December 2022, the gray bars represent the reproduction periods observed and monitored on the field. Copyright: @Marchessaux, CC BY 4.0 license.

The size at first maturity showed same values (males: 11.75 cm, females: 12.0 cm; Fig 6A). The temporal evolution of mature/immature stage frequency showed that matures males dominated significatively (ANOVA, Bonferroni post-hoc test, F = 84.6, p < 0.001) the population throughout the 61% of the time (Fig 6B). In contrast, mature females were observed only 33% of the time (Fig 6B) with matures females mainly observed between late summer and early fall. In fact, it appears that the population studied in the saltmarshes of Trapani is substantially composed by a population of young individuals. The temporal evolution of sex ratio Males:Females (M:F) showed highest values in September and October for both years (Fig 6C) indicating a dominance of males during these periods and a decrease in the number of females. For the rest of the time, the M:F sex ratio was close to 1 suggesting a relative equilibrium between both sex. The reproduction events (i.e. field observations of breeding couples face to face abdomens) were observed in July and September/October in 2021; and between May-July and in October for the year 2022 (Fig 6C). In the saltmarshes of Trapani, large matures males were always paired with large matures females (S4 Fig) illustrating here the reproduction capacity of large specimens only.

**Fig 7:**
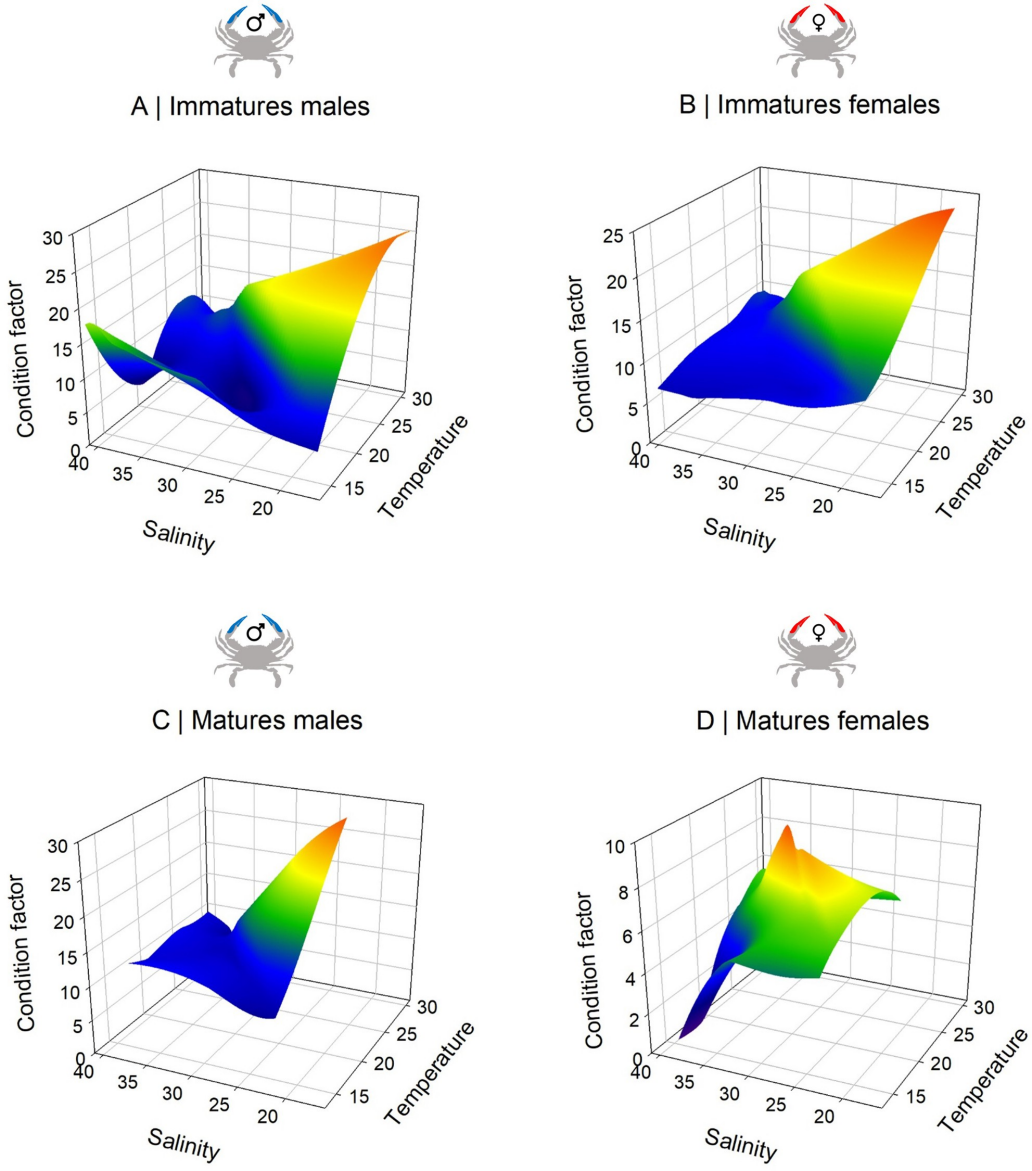
3-dimensional relationships between the condition factor K, temperature and salinity for (A) immatures males, (B) immatures females, (C) matures males and (D) matures females. The color gradient represents the values of the Fulton’s conditions factor (z axe). Copyright: @Marchessaux, CC BY 4.0 license.

The relation between the Fulton’s condition factor (K) and temperature/salinity showed different patterns between sexes and maturity stage (Fig 7). For both sexes immatures stages, the condition factor was high for highest temperatures (30°C) but lower salinity (S = 20) (Fig 7A and 7B). The same trends were observed for matures males where the condition factor was higher at high temperature (30°C) and at S = 25. In contrast, matures females showed an optimal condition factor for salinity S~32 and temperature at 22°C) (Fig 7C and 7D). Ovigerous females appeared in early autumn (September and October) for both study years (Table 2), and were sampled in the traps in the channel connecting saltmarshes to the sea. The ovigerous females CW ranged from 10.1 to 15.6 cm and the egg mass weight wet from 17 to 56 g representing an average of 25 ± 5% of the total female body wet weight (Table 2). The number of eggs estimated ranged from 985 137 (Female CW = 10.1 cm) to 2 156 874 eggs (Females CW: 13.3 cm) (Table 2). Two different eggs embryotic development stages were observed: 7 females with stage II-intermediate (orange), and 5 females with a stage III-pre-hatching (dark brown) (Table 2). The mean eggs diameter was significantly different (ANOVA, Bonferroni post-hoc test, F = 51.7, p < 0.002) between the stage II (209.4 ± 30.6 μm) and III (303.0 ± 4.3 μm).

**Table 2:** Fecundity of females *C*. *sapidus*: Sampling dates, female carapace width and wet weight, egg mass wet, total number of eggs estimated, eggs embryotic development stage (II: Intermediate; III: Pre-hatching), and mean (± standard deviation) egg diameter.

Sampling date	Female Carapace width (cm)	Female wet weight (g)	Egg mass wet weight (g)	Total number of eggs	Eggs embryotic development stage	Eggs diameter (μm ± SD)
2 Sept. 2021	11.0	168	42	1 769 297	II	241.1 ± 10.5
2 Sept. 2021	10.7	142	56	1 814 390	III	310.0 ± 14.5
2 Sept. 2021	13.2	147	36	1 278 276	II	197.9 ± 9.4
2 Sept. 2021	12.6	144	38	1 464 727	III	301.6 ± 16.1
6 Oct. 2021	10.9	91	21	1 621 248	II	231.2 ± 11.4
6 Oct. 2021	15.6	180	44	1 924 586	II	178.6 ± 8.5
6 Sept. 2022	11.9	87	19	1 675 254	III	298.5 ± 6.4
6 Sept. 2022	10.1	79	17	985 137	II	176.4 ± 8.7
6 Sept. 2022	10.4	90	22	1 053 546	III	301.7 ± 10.6
7 Oct. 2022	13.0	112	27	2 023 587	II	249.6 ± 4.6
7 Oct. 2022	13.3	90	20	2 156 874	III	303.2 ± 8.1
7 Oct. 2022	12.6	108	31	2 057 964	II	190.8 ± 4.5

## Discussion

**Fig 8:**
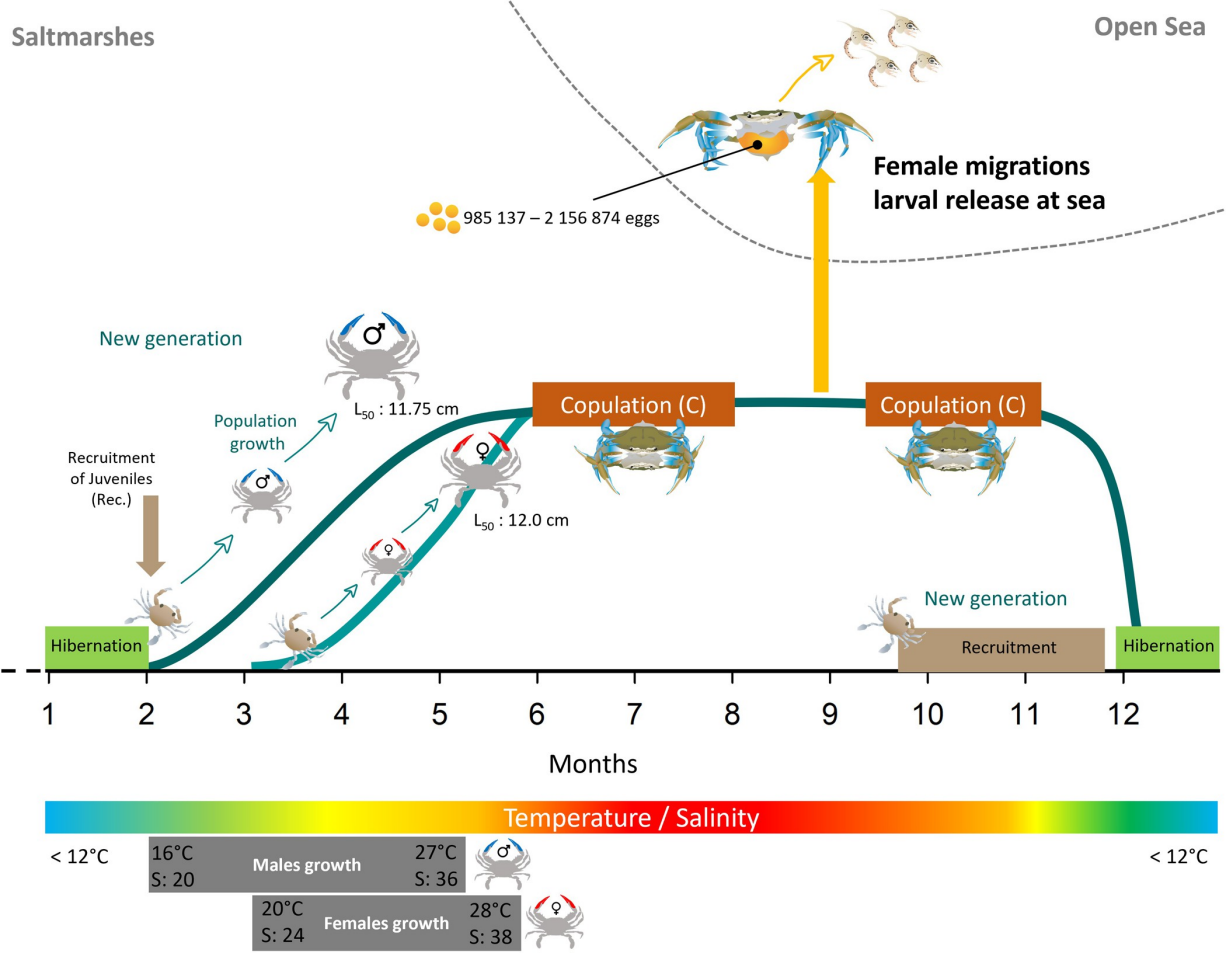
Schematic representation of the results obtained on the population size spectra dynamics in the saltmarshes of Trapani (Sicily, Italy). Copyright: @Marchessaux, CC BY 4.0 license.

Size spectra analysis is a powerful approach to describe the relationship between species size and abundance with environment [5]. Especially, in the case of invasive species, this approach in very useful to define the sensitives periods to implement management measures [5, 55]. By using this approach, our study demonstrates that the population of the invasive blue crab *C*. *sapidus* in the saltmarshes studied in Sicily is well-established consisting juveniles, subadults and adults. The analysis of the size spectra distribution showed integrative results on how environment was driving the blue crab’s population in term of growth, sexual maturity and copulation (Fig 8). In particular, we were able to provide more information on early stages (< 2 cm) that have been poorly studied because of the limited methodology to catch them (Fig 8).

### Population size structure

Our results on the morphometric relationships and allometric growth of blue crab were partially consistent with previous studies carried out in the Mediterranean basin. Comparing size spectra, blue crabs (males and females) showed positive allometry which is in disagreement with published studies in Turkey [56] for example. This may be because our study included the early juveniles’ stages (< 2 cm) in the allometric curves which is rarely presented in other studies. However, the positive allometry was in agreement with that presented for Egyptian [57] and Greek [58] blue crabs populations. The difference in the slopes of allometry equations was consistent with the fact that decapods and crustaceans exhibit significant plasticity in their phenotypic morphology that may be associated with environmental and/or genetic factors [59] such as time of year, temperature, food, stomach fullness, age differences, maturity stage, and sex [60, 61].

The largest crabs were caught in spring-summer and the smallest specimens in autumn-winter. While this pattern was not consistent with what observed in studies along the Atlantic coasts [62], but consistent with other populations in the Mediterranean Sea (e.g. Lesina Lagoon, Italy [63] and Monolimni Lagoon, Agean Sea [64]). Local conditions are indeed crucial factors in addressing local presence of species as showed by our analysis of size spectra. Indeed, CW-WW allometric slopes estimated in Trapani saltmarshes showed opposite relationships to temperature and salinity between males and females and this corroborates the hypothesis that are local environmental conditions that are mostly able to shape local presence. In our study, males showed a faster growth under cooler temperatures (spring) and salinities higher than 24, while females had a slower growth in spring and reached a maximum size in summer (Fig 8). Our findings were able to show that our study site could be an important recruitment zone of early stages (< 2 cm) whose were coming here to growth as observed in the native area in North Carolina (USA, native area) [65]. The recruitment period was identified in our study between September and November (Fig 8). The population’s growth period was identifier from the end of winter to early summer (Fig 8).

Our study in Trapani showed that *C*. *sapidus* has locally a complex life cycle, involving seasonal and spatially specific migrations for copulation and reproduction related to temperature and salinity [18, 22, 23, 26, 66, 67] thus generating spatio-temporal variability in population abundance at the small scale (Fig 8). In the saltmarshes, the population decreased in abundance and showed adult hibernation when water temperature was lower than 12°C (Fig 8) which is consistent both with what was observed by Marchessaux *et al*. [9] showing that this temperature represents the putative critical lower thermal threshold making local conditions unfavorable to the metabolism of *C*. *sapidus*, and with what is well known that specimens become inactive during col period [14], as reported in other Mediterranean and Atlantic regions [7, 68–70]. The relationship between Fulton’s conditions factor and temperature/salinity for immature and mature males and females showed different environmental windows explaining the complex life cycle of *C*. *sapidus*. Immature stages were present at salinities < 25 corresponding to winter salinities and a significant effect of temperature was observed. On the other hand, mature stages showed marked differences between males and females: temperature had a marked effect on the condition factor of males for salinities between 15 and 30, corresponding to winter and spring periods. On the contrary, the condition factor of females presented a more restricted environmental window with a thermal optimum of 24°C and a salinity of 30, observations consistent with the thermal tolerance of *C*. *sapidus* recently studied [9] where an optimum of 24°C was defined for the species’ metabolism. Our results showed a marked difference in environmental tolerance between males and females which could corroborated the idea of the complexity of the life cycle which occupies desalinated areas for reproduction and salty areas where the females mature their eggs. Thus, temperature and salinity play a key role in the maintenance of male and female populations throughout the year but also for sexual maturity, reproduction and the release of larvae at sea by the females. Indeed, mature females are catadromous, migrating from hyposaline waters (< 30 psu) to higher salinity waters to spawn [7, 71].

### Sexual maturity and reproduction

The sizes at sexual maturity (L_50_) estimated in our study at 11.75 cm for males and 12.0 cm for females were in the same range as those presented in the studies in native/introduced areas published (Table 3). However, it appeared that L_50_ differed locally. For example, in the native areas, females’ L_50_ was estimated at 15–16 cm (Florida [72]), 12–14.7 cm in Chesapeake Bay [73, 74], and 10.33 cm in Brazil [52] (Table 3). For male, L_50_ obtained in our study was larger than those cited in the literature in Brazil [69], but consistent with data available in the USA by Van-Engel (1958) [18] (Table 3). Our study showed that mature males were observed 2/3 of the time in contrast to mature females which were observed only 1/3 of the time mainly in summer, corresponding to the periods of maximum size observed in females. These observations were in accordance with the copulation period of *C*. *sapidus* characterized in our study, divided into two periods: late spring-early summer and early autumn when the highest percentage of matures males and females was observed. There are few data on the precise copulation periods of *C*. *sapidus* available in the literature and our results were in the same trends in the native areas in St. Johns River (Florida) and in the lower Chesapeake Bay [72, 75] with copulation observed during spring-summer. As determined by Jivoff [76], large matures males were always paired with large matures females, that is consistent with what we observed in our study. This strategy allows large males to be more competitive for access to females than small males, thus, large males win in aggressive interactions with rivals and transmit larger ejaculates to females [76]. The amount of time a male spends guarding influences female survival and access to her (this last phase is not clear). Male guarding time and ejaculate size increase when the male/female ratio is high, but ejaculate size decreases when males mate frequently, with short intervals between copulations [76]. In another hand, female blue crabs store sperm to fertilize the eggs they produce throughout their lives, where the reduced amounts of stored sperm may limit the total number of eggs they produce [76].

**Table 3:** Studies on the size at first maturity for *Callinectes sapidus*.

Country	Study site name	Native / Introduced	Sex	Size at maturity (cm)	Reference
Italy	Trapani saltmarshes	Introduced	Males	11.75	Our study
Brazil	Babitonga Bay	Native	Males	8.9	[69]
USA	Sarah’s Creek and Purtan Bay	Native	Males	10.7	[49]
USA	Chesapeake Bay	Native	Males	11.2	[18]
Italy	Trapani saltmarshes	Introduced	Females	12.00	Our study
Greece	Evros River	Introduced	Females	12.39	[64]
Turkey	Beymelek Lagoon	Introduced	Females	11.85	[81]
Brazil	Lagoon-Estuarine of Iguape and Cananéia	Native	Females	10.33	[52]
Brazil	Babitonga Bay	Native	Females	10.2	[69]
USA	St. Johns River	Native	Females	15–16	[72]
USA	Tampa Bay	Native	Females	13.0	[71]
USA	-	Native	Females	12.5	[101]
USA	Texas bay	Native	Females	12.0	[102]
USA	Chesapeake Bay	Native	Females	14.7	[73]
USA	Chesapeake Bay	Native	Females	12.0	[74]
USA	Chesapeake Bay	Native	Females	11.2	[18]

The variation in sex ratio could be explained by the different total mortality rates between sexes, the different migration patterns, and these parameters seemed to affect the males/females relative occurrence [77]. Especially, the sexual proportion of *Callinectes* species is related to reproductive behavior, female migrations, seasonal temperature variation, and salinity gradient [18, 78]. According to Berglund (1981) [77], males generally show a reduction in energy investment for growth in order to reduce pre-birth risk. In our study, the population was in favor of males which was in accordance with the high percentage of matures males all over the year which was consistent with similar studies in Italian [70, 79], Croatian [80] and Egyptian [57] lagoons, but diverged from sex ratio in a lagoon in southern Turkey, where the population was in favor of females [81]. A seasonal transition in sex ratio as a function of water temperature has been reported for *C*. *sapidus*, with a positive response in the case of male crab abundance, and negative in the case of females [82]. These conclusions were applicable to the results obtained in our study since the monthly variation of the sex ratio showed an almost total absence of females (dominance of males) at the end of summer (September-October) for both years studied, certainly due to the migration of adult females to waters of higher salinity, where they release their larvae [83]. This hypothesis was confirmed by the ovigerous females in the sea observed by fishermen (pers. obs.) and showing a high salinity tolerance/growth of females observed in the CW-WW slopes of our results.

### Spawning periods and female fecundity

Spawning of *C*. *sapidus* occurs throughout the year, with a predominance during the warmer months in its native sub-tropical range, pattern observed in most tropical brachyurans [52, 84, 85]. This reproductive pattern of *C*. *sapidus* can be classified as "seasonal-continuous" in Brazil, meaning a higher incidence in certain seasons of the year [86], possible because of the continuity of the physiological growth and reproductive process throughout the year. This pattern does not occur in temperate regions (native and non-native), where ovigerous females are present for a few months, being classified as discontinuous, which is related to the favorable environmental conditions that prevail during these periods [85]. This was the case in our study where ovigerous females were captured only in late summer/early autumn (September-October). These results were consistent with the results obtained in Italy in Lesina Lagoon [63] and in Northeast Aegean Sea [64]. This seaward migration is very important in the life cycle of the species, in which mature and ovigerous females move to higher salinity waters for gonad maturation. Saltwater provides lower larval mortality than waters with salinity variations; in addition, proposed benefits to marine larval development include reduced predation and increased survival in high salinity waters due to physiological limitations [52, 87–90]. Indeed, the blue crab’s salinity and temperature ranges for hatching fluctuate with geographic distribution and life history. Egg hatching generally proceeds between 19 and 29°C and for optimal salinity between 23 and 28 psu [91]. In our study, temperature and salinity were between 22.0 and 27.0°C and 32.5 and 37.3 respectively in late summer, period in which females were in advanced embryonic stages (II-intermediate and III-pre-hatching). Considering the embryonic stages of the eggs recorded in our study, it is certainly possible that the females were ovigerous earlier in the summer, as in the species’ native range [92], but that a high density migrated out of the study area before extruding the eggs onto the abdomen, which would explain the low number of ovigerous females captured in our study.

Additionally to the growth rates, sexual maturity, fecundity, associated with the number and diameter of eggs, gave an important information for estimating the reproductive potential of the species/population [74, 81]. Our results showed fecundity in the same range as that observed in the species native range in Chesapeake Bay (USA) [18] and Brazil [69] and in the Mediterranean Sea [93], but lower than populations from Mexico [94]. Such oscillations may be caused by several factors such as: the timing and stage of embryonic development of the eggs at the time of the count or the genetic characteristics of the populations [89]. However, our results remained consistent with the data available in the literature and provided new information on the fecundity of *C*. *sapidus* in Sicily, which has never been studied before.

## Conclusion

To conclude, *Callinectes sapidus* is known to have negative ecological impacts on invaded habitats in the Mediterranean Sea. Our study emphasized a novel approach to study an invasive species, in particular the blue crab *C*. *sapidus*. Sicily being an island does not have brackish lagoons or areas with large variations in salinity such as estuaries that are favorable for *C*. *sapidus* and where the species is mostly observed. Our study showed that the blue crab *C*. *sapidus* has succeeded in establishing itself in microhabitats such as saltmarshes in Sicily. In these areas, known to be favorable for the species in its native area [95–97], the blue crab exhibits a life cycle involving spatio-temporal migratory movements between saltmarshes and the adjacent coastal areas, which is consistent with its complex life cycle [7, 22–24, 68, 98]. These microhabitats provide favorable habitat for juvenile growth with extensive areas of *Ruppia maritima* and *Cymodocea nodosa* as observed in the Monolimni Lagoon [64]. These habitats are known to be productive and offer high abundances of macrozoobenthos [64]. On the other hand, we observed that large adults > 15 cm were rarely observed, which raises the hypothesis that the saltmarshes of Trapani would be a nursery area for the young stages of *C*. *sapidus*, which, once adult, would partially leave the saltmarshes in favor of other habitats such as the lagoon of Marsala where the species is observed in spring-summer (pers. obs.). The next step will be to determine the movements of adult crabs within the saltmarshes but also outside to confirm or not the hypothesis that the saltmarshes constitute a nursery area for *C*. *sapidus*. The results presented in this study provide evidence of the local adaptation of the species to invade different types of habitats, sometimes taking advantage of microhabitats such as saltmarshes, which would therefore be priority areas to study in Sicily.

Using a long-term and frequent population monitoring we were able to determine precisely how the environment (temperature and salinity) are influencing the males and females size spectra, combined with the periods of copulation and reproduction proved to be essential and useful to determine the periods suitable for the control of the species. Thus, thanks to our results the most sensitive periods for the species and therefore the periods when it is necessary to control the population should be in spring and summer when the highest percentage of mature males was recorded (more than 50% for 5 months). Females matured later and in smaller proportions (summer and autumn). Taking sexual maturity and the proportion of mature individuals into consideration, males would certainly be the key for an effective population control. But two hypotheses can be advanced. The first would be to fish intensively for males to reduce the sex ratio and prevent copulation. Intense, size-focused fishing of males may alter the male size structure and sex ratio of local populations (*sensu* Jivoff, 2003 [76]), especially for crustaceans species [99]. The second hypothesis is that harvesting mature females has the greatest impact on populations, and harvesting males is only effective if their numbers are such that females are unable to find a mate, as observed in North America [100]. These two hypothesis are to be explored in the near future. The most effective strategy would undoubtedly be to eliminate females before the period of sexual maturity and potential copulation. In our case, this would involve intensive control in spring.

### Copyright

All figures presented in this article and in the supplementary materials were produced by Marchessaux et al., under the copyright CC BY 4.0 license.

## Supporting information

S1 Fig Pictures of the traps specifically designed for our study with dimensions details, used to sample blue crabs. Copyright: @Marchessaux, CC BY 4.0 license.
(DOCX)

S2 Fig Temporal evolution of (A) temperature and (B) salinity between July 2021 and December 2022. Copyright: @Marchessaux, CC BY 4.0 license.
(DOCX)

S3 Fig Monthly evolution of the blue crab juveniles’ density (ind m^-2^) during the monitoring period. Copyright: @Marchessaux, CC BY 4.0 license.
(DOCX)

S4 Fig Relationships between immatures/matures males and females size (carapace width, cm) and linear regressions. Copyright: @Marchessaux, CC BY 4.0 license.
(DOCX)

S1 Table Characteristics of the habitat of the saltmarshes of Trapani. Areas of each substrate (m^2^) and percentage of coverage [%] [34, 45].
(DOCX)

## References

[1] MacIsaac HJ. Population structure of an introduced species (*Dreissena polymorpha*) along a wave-swept disturbance gradient. Oecologia. 1996;105: 484–492. 28307141 10.1007/BF00330011

[2] Sousa WP. Intertidal mosaics: patch size, propagule availability, and spatially variable patterns of succession. Ecology. 1984;65: 1918–1935.

[3] Hare MP, Nunney L, Schwartz MK, Ruzzante DE, Burford M, Waples RS, et al. Understanding and estimating effective population size for practical application in marine species management. Conserv Biol. 2011;25: 438–449. doi: 10.1111/j.1523-1739.2010.01637.x 21284731

[4] De Bie T, De Meester L, Brendonck L, Martens K, Goddeeris B, Ercken D, et al. Body size and dispersal mode as key traits determining metacommunity structure of aquatic organisms. Ecol Lett. 2012;15: 740–747. doi: 10.1111/j.1461-0248.2012.01794.x 22583795

[5] Petchey OL, Belgrano A. Body-size distributions and size-spectra: universal indicators of ecological status? The Royal Society; 2010. doi: 10.1098/rsbl.2010.0240 20444761 PMC2936225

[6] Nehring S. Invasion history and success of the American blue crab *Callinectes sapidus* in European and adjacent waters. In the wrong place-alien marine crustaceans: distribution, biology and impacts. Springer; 2011. pp. 607–624.

[7] Hines AH, Lipcius RN, Haddon AM. Population dynamics and habitat partitioning by size, sex, and molt stage of blue crabs *Callinectes sapidus* in a subestuary of central Chesapeake Bay. Mar Ecol Prog Ser. 1987;36: 55–64.

[8] Darnell MZ, Rittschof D, Darnell KM, McDowell RE. Lifetime reproductive potential of female blue crabs *Callinectes sapidus* in North Carolina, USA. Mar Ecol Prog Ser. 2009;394: 153–163.

[9] Marchessaux G, Bosch-Belmar M, Cilenti L, Lago N, Mangano MC, Marsiglia N, et al. The invasive blue crab *Callinectes sapidus* thermal response: Predicting metabolic suitability maps under future warming Mediterranean scenarios. Front Mar Sci. 2022;9: 1055404.

[10] Streftaris N, Zenetos A. Alien marine species in the Mediterranean-the 100 ‘Worst Invasives’ and their impact. Mediterr Mar Sci. 2006;7: 87–118.

[11] Mancinelli G, Bardelli R, Zenetos A. A global occurrence database of the Atlantic blue crab *Callinectes sapidus*. Sci Data. 2021;8: 1–10. 33863897 10.1038/s41597-021-00888-w PMC8052346

[12] Laughlin RA. Feeding habits of the blue crab, *Callinectes sapidus* Rathbun, in the Apalachicola estuary, Florida. Bull Mar Sci. 1982;32: 807–822.

[13] Hoeinghaus DJ, Zeug SC. Can stable isotope ratios provide for community-wide measures of trophic structure? comment. Ecology. 2008;89: 2353–2357. doi: 10.1890/07-1143.1 18724745

[14] Millikin MR. Synopsis of biological data on the blue crab, *Callinectes sapidus* Rathbun. National Oceanic and Atmospheric Administration, National Marine Fisheries …; 1984.

[15] Marchessaux G, Mangano MC, Bizzarri S, M’Rabet C, Principato E, Lago N, et al. Invasive blue crabs and small-scale fisheries in the Mediterranean sea: Local ecological knowledge, impacts and future management. Mar Policy. 2023;148: 105461.

[16] NOAA. Blue Crab Fishery Profile. 23 Jun 2023. Available: https://www.fisheries.noaa.gov/species/blue-crab#overview

[17] Churchill EP. Life history of the blue crab. Govt. print. off.; 1919.

[18] Van Engel WA. The blue crab and its fishery in Chesapeake Bay. Part 1. Reproduction, early development, growth and migration. Commer Fish Rev. 1958;20: 6.

[19] Cannarozzi L, Paoli C, Vassallo P, Cilenti L, Bevilacqua S, Lago N, et al. Donor-side and user-side evaluation of the Atlantic blue crab invasion on a Mediterranean lagoon. Mar Pollut Bull. 2023;189: 114758. doi: 10.1016/j.marpolbul.2023.114758 36867967

[20] Glamuzina L, Conides A, Mancinelli G, Glamuzina B. A Comparison of Traditional and Locally Novel Fishing Gear for the Exploitation of the Invasive Atlantic Blue Crab in the Eastern Adriatic Sea. J Mar Sci Eng. 2021;9: 1019.

[21] Mancinelli G, Chainho P, Cilenti L, Falco S, Kapiris K, Katselis G, et al. On the Atlantic blue crab (*Callinectes sapidus* Rathbun 1896) in southern European coastal waters: Time to turn a threat into a resource? Fish Res. 2017;194: 1–8. 10.1016/j.marpolbul.2017.02.050 28242280

[22] Aguilar R, Hines AH, Wolcott TG, Wolcott DL, Kramer MA, Lipcius RN. The timing and route of movement and migration of post-copulatory female blue crabs, *Callinectes sapidus* Rathbun, from the upper Chesapeake Bay. J Exp Mar Biol Ecol. 2005;319: 117–128.

[23] Jivoff PR, Hines AH, Quackenbush S. Reproduction and embryonic development. Blue Crab *Callinectes sapidus*. 2007.

[24] Epifanio CE. Early life history of the blue crab *Callinectes sapidus*: a review. J Shellfish Res. 2019;38: 1–22.

[25] Lipcius RN, Seitz RD, Seebo MS, Colón-Carrión D. Density, abundance and survival of the blue crab in seagrass and unstructured salt marsh nurseries of Chesapeake Bay. J Exp Mar Biol Ecol. 2005;319: 69–80.

[26] Hines AH, Wolcott TG, González-Gurriarán E, González-Escalante JL, Freire J. Movement patterns and migrations in crabs: telemetry of juvenile and adult behaviour in *Callinectes sapidus* and *Maja squinado*. J Mar Biol Assoc U K. 1995;75: 27–42.

[27] Perkins-Visser E, Wolcott TG, Wolcott DL. Nursery role of seagrass beds: enhanced growth of juvenile blue crabs (*Callinectes sapidus Rathbun*). J Exp Mar Biol Ecol. 1996;198: 155–173.

[28] Heck Jr KL, Hays G, Orth RJ. Critical evaluation of the nursery role hypothesis for seagrass meadows. Mar Ecol Prog Ser. 2003;253: 123–136.

[29] Lipcius RN, Eggleston DB, Heck Jr KL, Seitz RD, van Montrans J. Post-settlement abundance, survival, and growth of postlarvae and young juvenile blue crabs in nursery habitats. Blue Crab *Callinectes Sapidus* Md Sea Grant Coll Coll Park Md. 2007; 535–564.

[30] Shervette VR, Gelwick F, Hadley N. Decapod utilization of adjacent oyster, vegetated marsh, and non-vegetated bottom habitats in a Gulf of Mexico estuary. J Crustac Biol. 2011;31: 660–667.

[31] Posey MH, Alphin TD, Harwell H, Allen B. Importance of low salinity areas for juvenile blue crabs, *Callinectes sapidus* Rathbun, in river-dominated estuaries of southeastern United States. J Exp Mar Biol Ecol. 2005;319: 81–100.

[32] van Montfrans J, Ryer CH, Orth RJ. Substrate selection by blue crab *Callinectes sapidus* megalopae and first juvenile instars. Mar Ecol Prog Ser. 2003;260: 209–217.

[33] Vecchioni L, Russotto S, Arculeo M, Marrone F. On the occurrence of the invasive Atlantic blue crab *Callinectes sapidus* Rathbun 1896 (Decapoda: Brachyura: Portunidae) in Sicilian inland waters. Nat Hist Sci. 2022;9: 43–46.

[34] Bellino A, Mangano MC, Baldantoni D, Russell BD, Mannino AM, Mazzola A, et al. Seasonal patterns of biodiversity in Mediterranean coastal lagoons. Divers Distrib. 2019;25: 1512–1526.

[35] Messina G, Pezzino E, Montesanto G, Caruso D, Lombardo BM. The diversity of terrestrial isopods in the natural reserve “Saline di Trapani e Paceco”(Crustacea, Isopoda, Oniscidea) in northwestern Sicily. ZooKeys. 2012; 215. 10.3897/zookeys.176.2367 PMC3335416 22536110

[36] Weinstein MP. Shallow marsh habitats as primary nurseries for fishes and shellfish, Cape Fear River, North Carolina. Fish Bull. 1979;77: 339–357.

[37] Orth RJ, Vanmontfrans J. Utilization of a seagrass meadow and tidal marsh creek by blue crabs *Callinectes sapidus*. I. Seasonal and annual variations in abundance with emphasis on post-settlement juveniles. Mar Ecol Prog Ser. 1987;41: 283.

[38] Tupper M, Able K. Movements and food habits of striped bass (*Morone saxatilis*) in Delaware Bay (USA) salt marshes: comparison of a restored and a reference marsh. Mar Biol. 2000;137: 1049–1058.

[39] Rudershausen PJ, Merrell JH, Buckel JA. Factors Influencing Colonization and Survival of Juvenile Blue Crabs *Callinectes sapidus* in Southeastern US Tidal Creeks. Diversity. 2021;13: 491.

[40] Shenker JM, Dean JM. The utilization of an intertidal salt marsh creek by larval and juvenile fishes: abundance, diversity and temporal variation. Estuaries. 1979;2: 154–163.

[41] Schrandt M, Switzer T, Stafford C, Flaherty-Walia K, Paperno R, Matheson Jr R. Similar habitats, different communities: fish and large invertebrate assemblages in eastern Gulf of Mexico polyhaline seagrasses relate more to estuary morphology than latitude. Estuar Coast Shelf Sci. 2018;213: 217–229.

[42] Taylor DL, Fehon MM. Blue Crab (*Callinectes sapidus*) Population Structure in Southern New England Tidal Rivers: Patterns of Shallow-Water, Unvegetated Habitat Use and Quality. Estuaries Coasts. 2021;44: 1320–1343. 10.1007/s12237-020-00867-1 PMC8210731 34149332

[43] Ruas VM, Rodrigues MA, Dumont LFC, D’Incao F. Habitat selection of the pink shrimp Farfantepenaeus paulensis and the blue crab Callinectes sapidus in an estuary in southern Brazil: influence of salinity and submerged seagrass meadows. Nauplius. 2014;22: 113–125.

[44] Johnston CA, Caretti ON. Mangrove expansion into temperate marshes alters habitat quality for recruiting *Callinectes* spp. Mar Ecol Prog Ser. 2017;573: 1–14.

[45] Mannino AM, Sarà G. The effect of Ruppia cirrhosa features on macroalgae and suspended matter in a Mediterranean shallow system. Mar Ecol. 2006;27: 350–360.

[46] Sarà G, Romano C, Caruso M, Mazzola A. The new Lessepsian entry *Brachidontes pharaonis* (Fischer P., 1870)(Bivalvia, Mytilidae) in the western Mediterranean: a physiological analysis under varying natural conditions. 2000.

[47] Jivoff P. Sexual competition among male blue crab, *Callinectes sapidus*. Biol Bull. 1997;193: 368–380. 28574773 10.2307/1542939

[48] Pyle R, Cronin LE. The general anatomy of the blue crab, *Callinectes sapidus* Rathbun. Chesapeake Biological Laboratory; 1950.

[49] Van Engel WA. Development of the reproductively functional form in the male blue crab, *Callinectes sapidus*. Bull Mar Sci. 1990;46: 13–22.

[50] Olmi EJ III, Bishop JM. Variations in total width-weight relationships of blue crabs, *Callinectes sapidus*, in relation to sex, maturity, molt stage, and carapace form. J Crustac Biol. 1983;3: 575–581.

[51] Bland CE, Amerson HV. Occurrence and distribution in North Carolina waters of *Lagenidium callinectes* Couch, a fungal parasite of blue crab ova. Chesap Sci. 1974;15: 232–235.

[52] Severino-Rodrigues E, Musiello-Fernandes J, Mour ÁA, Branco GM, Canéo VO. Fecundity, reproductive seasonality and maturation size of *Callinectes sapidus* females (Decapoda: Portunidae) in the Southeast coast of Brazil. Rev Biol Trop. 2013;61: 595–602. 23885578 10.15517/rbt.v61i2.11162

[53] Kelly KL, Taylor CM. Effects of crude oil on survival and development in embryonated eggs in *Callinectes sapidus* Rathbun, 1896 (Decapoda, Portunidae). PeerJ. 2018;6: e5985. 30581659 10.7717/peerj.5985 PMC6294050

[54] Bagenal T. Methods for assessment of fish production in fresh waters-3. 1978.

[55] Buba Y, van Rijn I, Blowes SA, Sonin O, Edelist D, DeLong JP, et al. Remarkable size-spectra stability in a marine system undergoing massive invasion. Biol Lett. 2017;13: 20170159. doi: 10.1098/rsbl.2017.0159 28747531 PMC5543019

[56] Sangun L, Turelİ C, Akamca E, Duysak O. Width/length-weight and width-length relationships for 8 crab species from the north-eastern Mediterranean coast of Turkey. J Anim Vet Adv. 2009;8: 75–79.

[57] Razek FAA, Ismaiel M, Ameran MAA. Occurrence of the blue crab *Callinectes sapidus*, Rathbun, 1896, and its fisheries biology in Bardawil Lagoon, Sinai Peninsula, Egypt. Egypt J Aquat Res. 2016;42: 223–229.

[58] Kevrekidis T. Relative growth of the blue crab *Callinectes sapidus* in Thermaikos Gulf (Methoni Bay), northern Aegean Sea. Cah Biol Mar. 2019;60: 395–397.

[59] Maguire I, Marn N, Klobučar G. Morphological evidence for hidden diversity in the threatened stone crayfish *Austropotamobius torrentium* (Schrank, 1803)(Decapoda: Astacoidea: Astacidae) in Croatia. J Crustac Biol. 2017;37: 7–15.

[60] Bagenal TB. Age and growth. Methods Assess Fish Prod Fresh Waters. 1978; 101–136.

[61] Pauly D. Length-converted catch curves: a powerful tool for fisheries research in the Tropics (III: conclusion). Fishbyte. 1984;2: 9–10.

[62] Graham DJ, Perry H, Biesiot P, Fulford R. Fecundity and egg diameter of primiparous and multiparous blue crab *Callinectes sapidus* (Brachyura: Portunidae) in Mississippi waters. J Crustac Biol. 2012;32: 49–56.

[63] Cilenti L, Pazienza G, Scirocco T, Fabbrocini A, D’Adamo R. First record of ovigerous *Callinectes sapidus* (Rathbun, 1896) in the Gargano Lagoons (south-west Adriatic Sea). BioInvasions Rec. 2015;4.

[64] Kevrekidis K, Kevrekidis T, Mogias A, Boubonari T, Kantaridou F, Kaisari N, et al. Fisheries Biology and Basic Life-Cycle Characteristics of the Invasive Blue Crab *Callinectes sapidus* Rathbun in the Estuarine Area of the Evros River (Northeast Aegean Sea, Eastern Mediterranean). J Mar Sci Eng. 2023;11: 462.

[65] Etherington LL, Eggleston DB. Large-scale blue crab recruitment: linking postlarval transport, post-settlement planktonic dispersal, and multiple nursery habitats. Mar Ecol Prog Ser. 2000;204: 179–198.

[66] Carr SD, Tankersley RA, Hench JL, Forward RB Jr, Luettich Jr RA. Movement patterns and trajectories of ovigerous blue crabs *Callinectes sapidus* during the spawning migration. Estuar Coast Shelf Sci. 2004;60: 567–579.

[67] FORWARD RB, Cohen JH, Darnell MZ, Saal A. The circatidal rhythm in vertical swimming of female blue crabs, *Callinectes sapidus*, during their spawning migration: a reconsideration. J Shellfish Res. 2005;24: 587–590.

[68] Lipcius RN, Van Engel WA. Blue crab population dynamics in Chesapeake Bay: variation in abundance (York River, 1972–1988) and stock-recruit functions. Bull Mar Sci. 1990;46: 180–194.

[69] Pereira MJ, Branco JO, Christoffersen ML, Freitas F, Fracasso HAA, Pinheiro TC. Population biology of *Callinectes danae* and *Callinectes sapidus* (Crustacea: Brachyura: Portunidae) in the south-western Atlantic. J Mar Biol Assoc U K. 2009;89: 1341–1351.

[70] Mancinelli G, Carrozzo L, Costantini ML, Rossi L, Marini G, Pinna M. Occurrence of the Atlantic blue crab *Callinectes sapidus* Rathbun, 1896 in two Mediterranean coastal habitats: Temporary visitor or permanent resident? Estuar Coast Shelf Sci. 2013;135: 46–56.

[71] Steele P, Bert TM. Population ecology of the blue crab, *Callinectes sapidus* Rathbun, in a subtropical estuary: population structure, aspects of reproduction, and habitat partitioning. Fla Mar Res Publ USA. 1994.

[72] Tagatz ME. Growth of juvenile blue crabs, *Callinectes sapidus* Rathbun, in the St. Johns River, Florida. Fish Bull. 1968;67: 281–288.

[73] Prager MH, McConaugha JR, Jones CM, Geer PJ. Fecundity of blue crab, *Callinectes sapidus*, in Chesapeake Bay: biological, statistical and management considerations. Bull Mar Sci. 1990;46: 170–179.

[74] Rugolo LJ. Stock assessment of Chesapeake Bay blue crab (*Callinectes sapidus*). NOAA; 1997.

[75] Hines AH, Jivoff PR, Bushmann PJ, van Montfrans J, Reed SA, Wolcott DL, et al. Evidence for sperm limitation in the blue crab, *Callinectes sapidus*. Bull Mar Sci. 2003;72: 287–310.

[76] Jivoff P. A review of male mating success in the blue crab, *Callinectes sapidus*, in reference to the potential for fisheries-induced sperm limitation. Bull Mar Sci. 2003;72: 273–286.

[77] Berglund A. Sex dimorphism and skewed sex ratios in the prawn species Palaemon adspersus and P. squilla. Oikos. 1981; 158–162. 10.1007/BF00347589 28309987

[78] Tagatz ME. The Fishery for Blue Crabs in the St. Johns River, Florida with Special Reference to Fluctuation in Yield Between 1961 and 1962. US Department of the Interior, Fish and Wildlife Service; 1965.

[79] Carrozzo L, Potenza L, Carlino P, Costantini ML, Rossi L, Mancinelli G. Seasonal abundance and trophic position of the Atlantic blue crab *Callinectes sapidus* Rathbun 1896 in a Mediterranean coastal habitat. Rendiconti Lincei. 2014;25: 201–208.

[80] Dulčić J, Conides A, Onofri V, Matić-Skoko S, Glamuzina B. The occurrence of the blue crab, *Callinectes sapidus* Rathbun, 1896 (Decapoda, Brachyura, Portunidae) in the eastern Adriatic (Croatian coast). Crustaceana. 2008;81: 403–409.

[81] Sumer C, Teksam I, Karatas H, Beyhan T, Aydin CM. Growth and reproduction biology of the blue crab, *Callinectes sapidus* Rathbun, 1896, in the Beymelek Lagoon (Southwestern Coast of Turkey). Turk J Fish Aquat Sci. 2013;13: 675–684.

[82] Harding JM, Mann R. Observations of distribution, size, and sex ratio of mature blue crabs, *Callinectes sapidus*, from a Chesapeake Bay tributary in relation to oyster habitat and environmental factors. Bull Mar Sci. 2010;86: 75–91.

[83] Branco JO, Masunari S. Reprodutive ecology of the blue crab, *Callinectes danae* Smith, 1869 in the Conceição Lagoon system, Santa Catarina Isle, Brazil. Rev Bras Biol. 2000;60: 17–27. 10838920 10.1590/s0034-71082000000100004

[84] Eggleston DB, Johnson EG, Hightower JE. Population dynamics and stock assessment of the blue crab in North Carolina. 2004.

[85] Rasheed S, Mustaquim J. Size at sexual maturity, breeding season and fecundity of three-spot swimming crab *Portunus sanguinolentus* (Herbst, 1783)(Decapoda, Brachyura, Portunidae) occurring in the coastal waters of Karachi, Pakistan. Fish Res. 2010;103: 56–62.

[86] Pinheiro MAA, Fransozo A. Reproduction of the speckled swimming crab *Arenaeus cribrarius* (Brachyura: Portunidae) on the Brazilian coast near 23 30′ S. J Crustac Biol. 2002;22: 416–428.

[87] Buchanan BA, Stoner AW. Distributional patterns of blue crabs (*Callinectes sp*.) in a tropical estuarine lagoon. Estuaries. 1988;11: 231–239.

[88] Tankersley RA, Wieber MG, Sigala MA, Kachurak KA. Migratory behavior of ovigerous blue crabs *Callinectes sapidus*: evidence for selective tidal-stream transport. Biol Bull. 1998;195: 168–173. 28570184 10.2307/1542824

[89] Dickinson GH, Rittschof D, Latanich C. Spawning biology of the blue crab, *Callinectes sapidus*, in North Carolina. Bull Mar Sci. 2006;79: 273–285.

[90] Ortiz-Leon HJ, Ad J-N, Cordero ES. Temporal and spatial distribution of the crab *Callinectes sapidus* (Decapoda: Portunidae) in Chetumal Bay, Quintana Roo, Mexico. Rev Biol Trop. 2007;55: 235–245. 18457132

[91] Sandoz M, Rogers R. The Effect of Environmental Factors on Hatching, Moulting, and Survival of Zoea Larvae of the Blue Crab *Callinectes Sapidus* Rathbun. Ecology. 1944;25: 216–228. doi: 10.2307/1930693

[92] Ogburn MB, Habegger LC. Reproductive status of *Callinectes sapidus* as an indicator of spawning habitat in the South Atlantic Bight, USA. Estuaries Coasts. 2015;38: 2059–2069.

[93] Türeli C, Yeşilyurt İN, Nevşat İE. Female reproductive pattern of *Callinectes sapidus* Rathbun, 1896 (Brachyura: Portunidae) in Iskenderun Bay, Eastern Mediterranean. Zool Middle East. 2018;64: 55–63.

[94] Hsueh P-W. Factors affecting the population dynamics of the lesser blue crab (*Callinectes similis* Williams) in barrier island salt marsh habitats of the Gulf of Mexico. J Ala Acad Sci. 1992;63: 1–9.

[95] Fitz HC, Wiegert RG. Utilization of the intertidal zone of a salt marsh by the blue crab *Callinectes sapidus*: density, return frequency, and feeding habits. Mar Ecol Prog Ser. 1991; 249–260.

[96] Fitz HCI. The utilization of a salt marsh estuary by the blue crab, *Callinectes sapidus*. 1992.

[97] Wrona AB. Determining movement patterns and habitat use of blue crabs (*Callinectes sapidus Rathbun*) in a Georgia saltmarsh estuary with the use of ultrasonic telemetry and a geographic information system (GIS). PhD Thesis, University of Georgia. 2004.

[98] Turner HV, Wolcott DL, Wolcott TG, Hines AH. Post-mating behavior, intramolt growth, and onset of migration to Chesapeake Bay spawning grounds by adult female blue crabs, *Callinectes sapidus* Rathbun. J Exp Mar Biol Ecol. 2003;295: 107–130.

[99] Ogburn MB. The effects of sex-biased fisheries on crustacean sex ratios and reproductive output. Invertebr Reprod Dev. 2019;63: 200–207.

[100] Rains SA, Wilberg MJ, Miller TJ. Evaluation of fishery-induced sperm limitation in Chesapeake Bay blue crab using an individual-based model. Mar Ecol Prog Ser. 2018;596: 127–142.

[101] Guillory V, Hein S. Sexual maturity in blue crabs, *Callinectes sapidus*. Proceeding Coast Fish Louisiana’s Blue Crab Resour St Univ Acad Sci. 1997;59: 5–7.

[102] Fisher MR. Effect of temperature and salinity on size at maturity of female blue crabs. Trans Am Fish Soc. 1999;128: 499–506.

