# Supplementary material for: Environmental drivers of size-based population structure, sexual maturity and fecundity: A study of the invasive blue crab Callinectes sapidus (Rathbun, 1896) in the Mediterranean Sea
Source: PLoS One. 2023 Aug 7;18(8):e0289611. doi: 10.1371/journal.pone.0289611 (PMC10406326; doi:10.1371/journal.pone.0289611)
Supplement: S2 Fig — Temporal evolution of (A) temperature and (B) salinity between July 2021 and December 2022. Copyright: @Marchessaux, CC BY 4.0 license. (DOCX) [file pone.0289611.s002.docx]

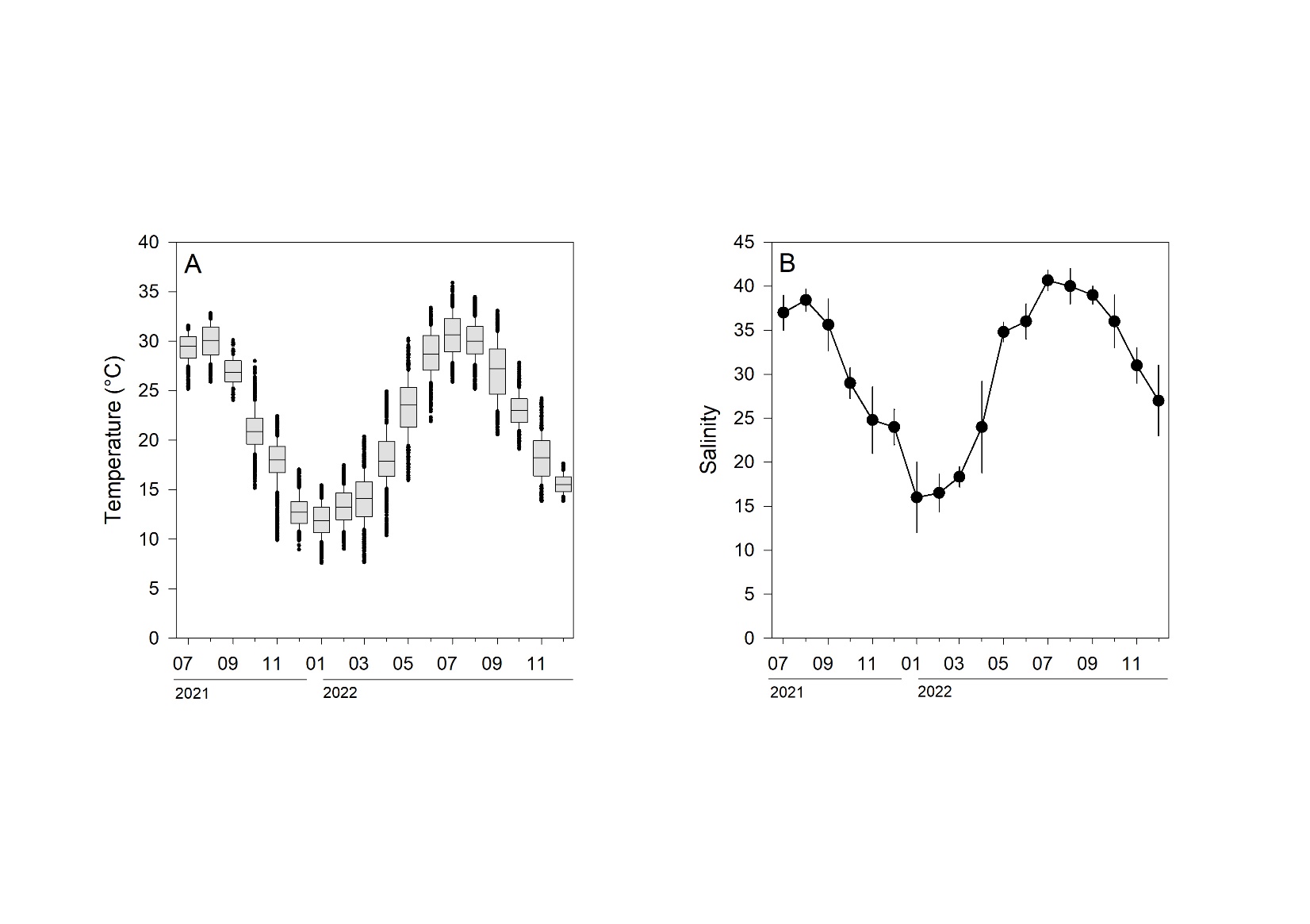


**Supplementary Figure 2.** Temporal evolution of (A) temperature and (B) salinity between July 2021 and December 2022. Copyright: @Marchessaux, CC BY 4.0 license.
