# Supplementary material for: Environmental drivers of size-based population structure, sexual maturity and fecundity: A study of the invasive blue crab Callinectes sapidus (Rathbun, 1896) in the Mediterranean Sea
Source: PLoS One. 2023 Aug 7;18(8):e0289611. doi: 10.1371/journal.pone.0289611 (PMC10406326; doi:10.1371/journal.pone.0289611)
Supplement: S4 Fig — Copyright: @Marchessaux, CC BY 4.0 license. (DOCX) [file pone.0289611.s004.docx]

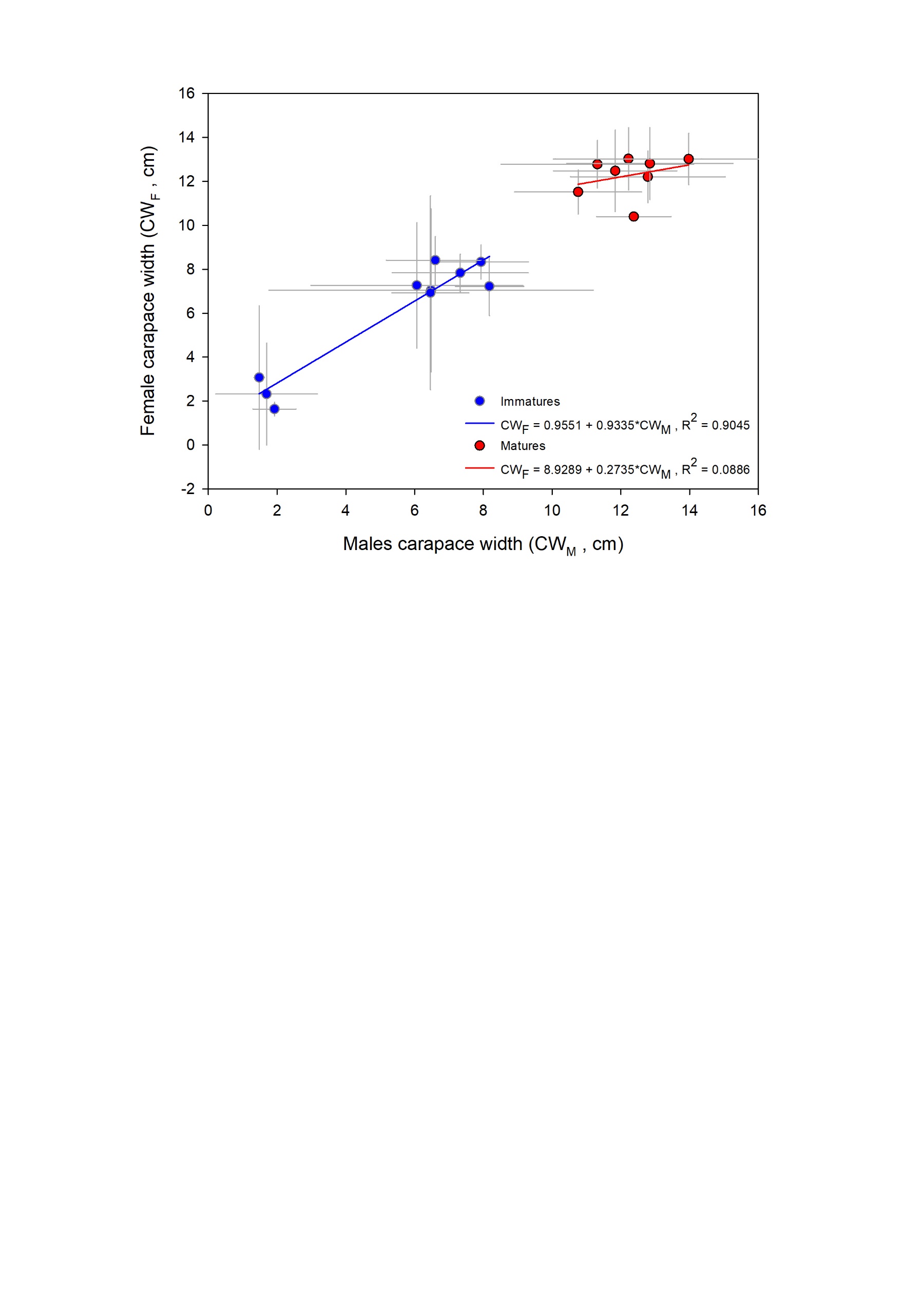


**Supplementary Figure 4.** Relationships between immatures/matures males and females size (carapace width, cm) and linear regressions. Copyright: @Marchessaux, CC BY 4.0 license.
