# Supplementary material for: Environmental drivers of size-based population structure, sexual maturity and fecundity: A study of the invasive blue crab Callinectes sapidus (Rathbun, 1896) in the Mediterranean Sea
Source: PLoS One. 2023 Aug 7;18(8):e0289611. doi: 10.1371/journal.pone.0289611 (PMC10406326; doi:10.1371/journal.pone.0289611)
Supplement: S1 Table — Areas of each substrate (m2) and percentage of coverage [%] [34, 45]. (DOCX) [file pone.0289611.s005.docx]

**Supplementary Table 1.** Characteristics of the habitat of the saltmarshes of Trapani. Areas of each substrate (m^2^) and percentage of coverage [%].

| Saltmarshe ID | Total area  (m^2^) | Sandy-muddy  (m^2^) [%] | *Ruppia maritima*  (m^2^) [%] | *Cymodocea nodosa* (m^2^) [%] |
| --- | --- | --- | --- | --- |
| Salmarsh S1 | 3 229 | 1 575 [49] | 1 654 [51] | - |
| Salmarsh S2 | 1 386 | 1 386 [100] | - | - |
| Salmarsh S3 | 3 412 | 2 370 [69] | - | 1 042 [31] |
| Salmarsh S4 | 2 449 | 1 005 [41] | 1 444 [59] | - |
| Salmarsh S5 | 1 540 | 54 [4] | - | 1 486 [96] |
| Salmarsh S6 | 4 262 | 4 128 [97] | - | 134 [3] |
| Salmarsh S7 | 7 399 | 6 215 [84] | 1 183 [16] | - |
| Salmarsh S8 | 4 187 | 3 266 [78] | - | 912 [22] |
| Salmarsh S9 | 824 | 824 [100] | - | - |
